# Capacity and readiness of civil society organisations to implement community case management of malaria in Kenya

**DOI:** 10.11604/pamj.supp.2016.25.2.9305

**Published:** 2016-11-26

**Authors:** Enock Marita, Jared Oule, Margaret Mungai, Sylla Thiam, Festus Ilako

**Affiliations:** 1 Amref Health Africa in Kenya; 2 Amref Health Africa in West Africa; 3 Amref Health Africa Headquarters

**Keywords:** Capacity assessment, risk management, malaria case management, civil society organisations, capacity building, governance

## Abstract

**Introduction:**

Civil Society Organizations (CSOs) contribute to achieving development goals through advocacy, social mobilisation and provision of health services. CSO programming is a key component of Global Fund (GF) grants; however, CSOs face technical and governance capacity challenges in grant utilisation leading to missed opportunities for improving health at community level. Amref Health Africa was appointed Principal Recipient of a GF grant aimed at scaling up community case management of malaria through CSOs as sub-recipients in western Kenya. To identify potential risks and strengthen grant management, Amref Health Africa and the Ministry of Health conducted a capacity needs assessment to determine the capacity of CSOs to effectively utilise grants.

**Methods:**

26 selected CSOs participated in this study. Document reviews and on-site assessments and observations were conducted using structured tool. The five main assessment areas were: governance and risk management; strategic and operational planning; monitoring and evaluation; programme management; and financial management. Overall performance was grouped into four categories: 3.0-2.5 (excellent), 2.0-2.4 (good), 1.5-1.9 (fair), and 1.0-1.4 (poor). Data were collected and analysed using Excel software.

**Results:**

Twenty five out of 26 CSOs were legally compliant. 14(54%) CSOs were categorized as good; 7(27%) as excellent; 3(12%) as poor and 2(8%) as fair. Most CSOs had good programme management capacity but monitoring and evaluation presented the most capacity gaps.

**Conclusion:**

More than 75% of the CSOs were rated as excellent or good. A capacity building plan, programme risk management plan and oversight mechanisms were important for successful grant implementation.

## Introduction

It is widely recognized that Civil Society Organizations (CSOs) play an important role in social, political and economic development of nations. CSOs also play a vital role in regional, sub-regional, national and local processes to strengthen governance and transparency, and hold governments accountable in the attainment of development goals. CSOs have the comparative advantage of having non-bureaucratized and responsive structures and a willingness to address sensitive social issues [1]. Grass-roots efforts by CSOs were instrumental in the creation of the Global Fund. CSOs implement Global Fund-supported programmes and play an integral role in resource mobilization, advocacy and policy dialogue. In the Global Fund context, “civil society includes non-governmental organizations, community groups, faith-based organizations, foundations, advocacy groups and networks of people living with diseases - essentially all those communities that are neither government nor profit-seeking enterprises” [2]. CSOs play an important role in governance of the Global Fund. Of the twenty voting seats on the Global Fund Board, three are allocated to CSOs: one for non-governmental organizations in the developed world, one for non-governmental organizations in the developing world and most importantly, one seat representing communities living with the three diseases addressed by the Global Fund: malaria, human immunodeficiency virus (HIV) and tuberculosis (TB) [2]. Inclusion of civil society in Global Fund Board deliberations helps keep the reality of these three diseases in the forefront of discussions. CSOs take part in deliberations at national level, sitting on Country Coordinating Mechanisms, where they help set the country’s priorities in the management of the three diseases, alongside other stakeholders. Challenges remain to ensure meaningful participation by CSOs in all [2]. Implementing programmes (including malaria control programmes) at community level is another area where CSOs have proven to be effective as social mobilizers, advocates and service providers, serving as Principal Recipients and sub-recipients [2]. In particular, CSOs play a key role in reaching out to affected key populations not usually reached by services, such as people who inject drugs, men who have sex with men, villagers with poor access to health facilities and sex workers [2, 3]. CSOs are increasingly implementing interventions that encourage sustainability of responses, empower key populations and promote health, social and structural changes in the fight against diseases especially malaria, HIV and TB [2, 3]. In Kenya, there are about 554 registered CSOs in the health sector, of which 110 are involved in malaria control [4]. Many CSOs do not have stable funding sources and rely on unpredictable, donor-driven project funding. Chronic limited human resource capacity, inability to recruit and retain high quality staff and high staff turnover are other challenges faced by CSOs [1].

To date, national efforts in malaria control in endemic areas of Africa have focused on scaling-up the availability and use of long-lasting insecticidal nets (LLINs), indoor residual spraying (IRS) with insecticides, intermittent preventive treatment during pregnancy (IPTp), and diagnostic testing and treatment of confirmed uncomplicated malaria using artemisinin-based combination therapy (ACT) [5]. The World Malaria Report 2010 noted that the increase in international funding commitments had allowed a massive scale up of malaria control interventions in many countries, along with sometimes dramatic reductions in malaria burden [6]. A community-based approach is important to enable governments to match resources to local burden, and allow affected communities to take a more aggressive approach to lowering and ultimately eliminating transmission in malaria endemic areas [5]. Since 2001, community-based case management of malaria (CCMM), a strategy formerly known as ‘home management of malaria’, in which antimalarial treatment is made available close to the home by community health volunteers, has been a cornerstone of the WHO-recommended strategy to improve access to prompt, effective malaria treatment, especially in remote, underserved areas with high malaria transmission [7]. CCMM has been shown to be effective in reducing mortality and morbidity [8–10] and could be a useful addition to achieving sustained malaria control, a prerequisite for malaria elimination. Rapid malaria elimination in areas of high transmission potential will require large-scale, innovative preventive and curative interventions that will reach all populations at risk. Investing in helping communities to become self-reliant and increasing the demand for malaria control in affected communities could improve the sustainability of the required high coverage of key interventions [5]. Global Fund Round 10 provided a grant to support the CCMM strategy to be implemented partly through CSOs in Kenya. The risks associated with inadequate capacity among CSOs are huge in terms of grant misuse and misapplication. To improve grant performance and develop risk mitigation measures, Amref Health Africa conducted a baseline capacity and readiness needs assessment to serve as a basis for decision making on contracting CSOs to implement the grant. The aim of the capacity and readiness assessment was to establish baseline capacities and identify capacity gaps among 26 CSOs pre-selected to implement the Global Fund Malaria Round Ten Grant at community level and to inform a capacity strengthening plan and risk mitigation measures.

## Methods

**Study location:** the study was carried out in ten counties in the former Nyanza and Western Provinces of Kenya. The counties were Kisii, Nyamira, Migori, Homa Bay, Kisumu, Siaya, Busia, Bungoma, Kakamega and Vihiga. The study area has epidemic and holoendemic malaria with a prevalence of about 38% [11] and has a number of CSOs involved in social and economic development. The study was done in March, 2013.

**Study design:** this was a cross-sectional descriptive study. The CSOs were subjected to a comprehensive organizational capacity assessment using a standardised organisational capacity assessment (OCA) tool as outlined below.

### Desk review of source documents

The Expressions of Interest (EOIs) from the 26 pre-selected CSOs were reviewed to assess on-site capacity in relation to the objectives, service delivery areas, targets, implementation strategies and budget of the Global Fund Round 10 Malaria Component proposal.

### On-site assessments and observations

The study team visited the head offices and some of the regional offices of the pre-selected CSOs to assess their capacity through reviewing documents and interviewing relevant staff. The assessment was carried out in two parts: verification of the information given in the expressions of interest (EoI) document and assessment of governance and risk management; strategic and operational planning; programme management; financial management and reporting; and monitoring and evaluation through examining documentary evidence of the legal existence of the organisation; ability to keep to contractual obligations under other projects; membership and functioning of boards or management committees; and the extent to which the oversight body is able to manage risk and ensure accountability. Strategic and operational planning was assessed by checking the availability of well-developed strategic plans and annual operational plans. Assessment of a good mix of partnerships and income streams to support the sustainable operations of the organization was made. Programme management and implementation capacity (reporting, record keeping, human resources policy, staffing and supervision) were assessed to establish the capacity to implement projects, develop coherent work plans and undertake proposed activities effectively. Where possible, project reports, specific project audits and reports on performance from donors was reviewed to validate how effectively prior grants had been utilised. Financial management systems were assessed and verified, including staffing of finance units, systems for financial controls and accounting, financial monitoring and reporting. The tools, systems and processes for monitoring activities, verifying gathered data and reporting to relevant stakeholders including funding agencies were assessed. Based on the findings, each of the five (5) assessment areas were given a weighted score and the total for all the sections calculated to arrive at a numerical weighted average between 1 and 3. Any CSO falling in the range of 2.5-3.0 was categorized as Excellent (strong capacity with minor gaps); 2.0 - 2.4 were categorized as Good (can manage and implement grants but need significant short term capacity building); 1.5-1.9 as Fair (have capacity gaps that would present accountability risks to any disbursed funds and require major capacity building); and 1.0 - 1.4 as Poor (have major capacity gaps that would present high accountability risks to any disbursed funds), see Table 1. The opinions, views, observations and data obtained were transcribed, grouped and coded depending on the topic and analysed using Excel software.

**Table 1:** Classification of scores

	**Colour code**	**Score range**	**Requirement for capacity building**	**Level of Risk**
1	Excellent	2.5-3.0	Minimal or no capacity building	Low risk
2	Good	2.0-2.4	Moderate capacity building	Moderate risk
3	Fair	1.5-1.9	High capacity building efforts	High risk
4	Poor	1.0-1.4	Extensive capacity building	Serious risk

Classification of scores. Findings of Marita *et al.,* capacity and readiness of CSOs to implement community case management of malaria study, Kenya, 2016.

**Sampling design and sample size:** the Ministry of Health in conjunction with Amref Health Africa selected CSOs based on their proposals. A total of 26 out of 543 CSOs were selected and were assessed for their capacity and readiness to implement the grant. All the organizations must have had operations in former Nyanza and Western provinces where the project was to be implemented.

**Study instruments:** a structured checklist was used to collect data.

**Data management and analysis:** data were entered into Excel, cleaned and analysed. Categorisation of data was made according to each thematic area. Descriptive analyses, frequencies, proportions of observations were documented.

## Results

### Categories of CSOs assessed

**Figure 1:**
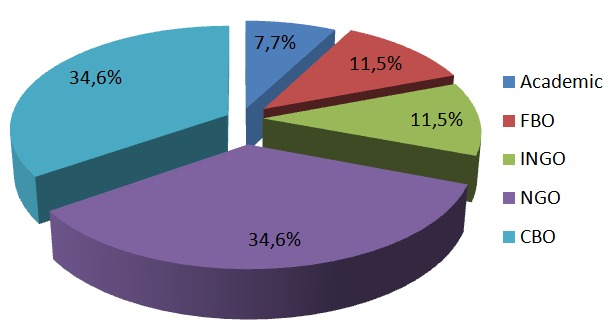
Categories of Civil Society Organizations

The 26 CSOs were classified into five categories: 2(7.7%) were academic institutions; 3(11.5%) were faith-based organisations (FBO); 3(11.5%) were international non-governmental organisations (INGO); 9(34.6%) were local non-governmental organisations (LNGO); and 9(34.6%) community-based organisations (CBO), Figure 1. There was wide diversity among CSOs in terms of activities, size and reach, ranging from small organizations run by volunteers operating at community level, to organizations with large budgets and professional staff with activities throughout the East African region.

### Legal compliance of CSOs

Only 1/26 (4%) CSOs did not provide adequate information about its full profile. This CSO had neither the documentation, staff, nor experience to implement the proposed activities in the target area. All the other 25 (96%) CSOs assessed had evidence of formal registration or exemption, with registration status either current or renewal applied for. Most organizations had boards of directors or trustees either sitting in Kenya for local CSOs or overseas for international non-governmental organisations. Some organisations had advisory boards which worked with the secretariat at the planning stages to develop objectives and strategies for the management teams. The frequency of board meetings or senior management committee meetings varied from once a month to once a year, although in some board membership did not comply with usual standards. Some board members doubled up as the secretariat of the CSOs. Generally the most critical data provided by the CSOs in their expression of interest (EOIs) was valid. All CSOs except one met their statutory obligations including providing National Social Security Fund (NSSF) and Pay as You Earn (PAYE) for her staff.

### Findings of on-site assessments and observations

Based on the overall assessment, 7(27%) CSOs scored as excellent, 14(54%) as good, 2(8%) as fair and 3(12%) as poor. The INGOs and LNGOs were mainly scored Excellent or Good. The three FBOs assessed were each rated excellent, good and poor. Majority of CBOs 5(56%) were in Good category and 2(22%) each rated fair and poor, see Table 2.

**Table 2:** Summary of CSOs ratings/score

Type of CSO	Percentage rated in each category				
	Excellent (2.5-3.0)	Good (2.0-2.4)	Fair (1.5-1.9)	Poor (1.0-1.4)	Total
Academic	50 (1)	50 (1)	0	0	100 (2)
CBO	0	56 (5)	22 (2)	22 (2)	100 (9)
FBO	33 (1)	33 (1)	0	33 (1)	100 (3)
INGO	33(1)	67 (2)	0	0	100 (3)
NGO	44 (4)	56 (5)	0	0	100 (9)
All CSOs	27 (7)	54 (14)	8 (2)	12 (3)	100 (26)

Summary of CSOs ratings/score. Findings of Marita *et al.,* capacity and readiness of CSOs to implement community case management of malaria study, Kenya, 2016.

### Findings of management context of the CSOs

**Figure 2:**
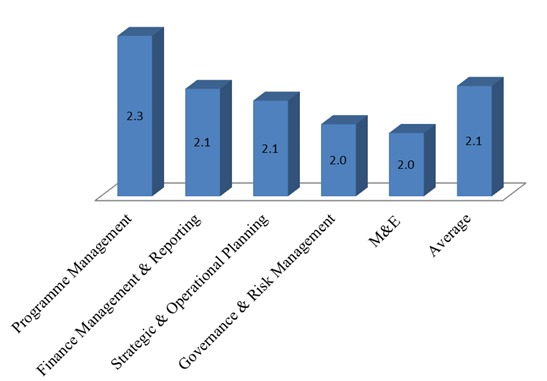
Overall Civil Society Organizations score for management categories

From the assessment on the five major management contexts, the CSOs scored an average of 2.1 which was graded as Good. Of the five categories, Governance and Risk Management and M&E scored least at 2.0, with program management scoring the highest at 2.3 (see Figure 2).

***Governance and risk management:*** some factors that negatively affected the scores under governance and risk management included: board membership not an odd number 4(15.4%); replacement or election of board members irregular or no evidence of change in board membership 5(19.2%); no clear separation of roles between the board and secretariat 3(11.5%); documentation of board meetings not supported with evidence such as minutes 6(23.1%); no regular annual general meetings 8(30.7%); some board member(s) unduly dominating activities 2(7.7%).

***Strategic and operational planning:*** only 2(7.7%) CSOs scored poorly with 1.2 and 1.3 respectively. However some deficiencies were observed, including: work plans not comprehensive 5(19.2%); no clear objectives 4(15.4%); no strategic plan or poorly elaborated strategic plans 11(42.3%); activities and targets not well stated.

***Programme management:*** only 2(7.7%) CSOs scored below average.

**Deficiencies under program management included:** delayed reporting or poor performance on reporting and poor record keeping 3(11.5%); lack of a human resources policy 6(23.1%); inadequate staffing 5(19.2%); programmes administered from the secretariat in Nairobi with gaps in supervision at field level 4(15.4%).

***Finance management and reporting:*** finance management and reporting showed the most deficiencies including: inadequate segregation of roles 10(38.4%); a weak finance department operated by volunteers or part time staff lacking academic qualifications, or no dedicated finance staff 5(19.2%); financial systems with insufficient controls to support effective resource allocation and accounting 9(34.6); poor budgetary controls 12(46%); no audited accounts or no focused internal audits 8(30.7%); petty cash thresholds too high or no petty cash accounts 6(23.1%); financial advisors doubling up as members of the auditing firm and inadequately skilled external audit team too 2(7.7%); cash flow projections not prepared 6(23.1%); statutory obligations such as PAYE, NHIF not honoured 7(26.9%); not meeting procurement requirements or procurement processes unclear or lengthy 12(46.1%).

***Monitoring and evaluation risks (M&E):*** four (15.4%) CSOs scored below average for M&E capabilities. Deficiencies included: limited or no M&E capacity due to lack of qualified staff 14(53.8%); engagement of consultants on a volunteer basis 3(11.5%); field officers acting as M&E officers; unavailability of an organizational M&E manual 16(61.5%); unclear field monitoring of activities.

### Capacity gaps

**Figure 3:**
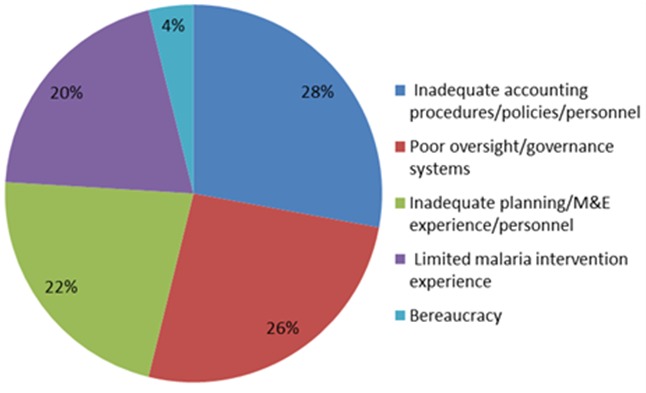
Major weaknesses of Civil Society Organizations

The CSOs had various capacity gaps which were grouped into five main areas: inadequate accounting procedures/policies/personnel; poor oversight/governance systems; inadequate planning/M&E experience/personnel; limited malaria intervention experience; bureaucracy (un-clear/delayed decision making processes). Inadequate accounting procedures, policies and personnel were the leading cause for concern followed by governance issues, while the least was bureaucratic hindrances especially among the academic CSOs, see Figure 3.

### Identified Strengths of the CSOs

**Figure 4:**
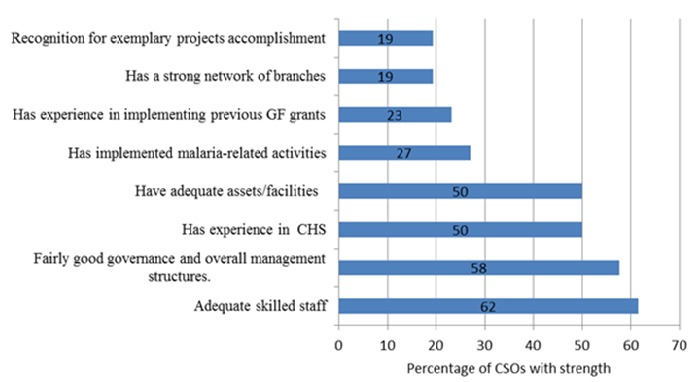
Strengths of Civil Society Organizations

Despite the weaknesses identified the CSOs had a number of strengths that could contribute to the success of grant management. These were categorized into eight main areas: adequate skilled staff; adequate governance and overall management structures; experience in community health strategies; adequate assets and facilities; previous implementation of malaria-related activities; experience in implementing previous GF grants; strong networks of branches; recognition of previous exemplary project accomplishments. Most CSOs had adequate skilled staff and adequate overall management, possessed assets or facilities necessary to implement the grant and were experienced in community health strategies. Some CSOs had experience in implementing previous GF grants or experience in managing malaria related activities. A few CSOs had a strong network of branches in the areas of implementation. Some CSOs had been recognised for their exemplary work accomplishment, see Figure 4.

## Discussion

At a presentation given during an East African Association of Grantmakers (EAAG) meeting in Uganda, Kirongo reported gaps in current Kenya Legal and regulatory framework for CSOs. These included multiple and overlapping legal and regulatory regimes presenting difficulties for those seeking a harmonized reporting framework; difficulties in monitoring compliance and accountability of CSOs; current CSO laws being centralised in Nairobi presenting challenges to the spirit of devolution as enshrined in the new constitution; inadequate mechanisms and processes for self-regulation for CSOs in Kenya; current laws for CSOs not explicitly addressing leadership and integrity issues as provided for in the Constitution; absence of an independent body to provide checks and balances in cases where the self-regulatory mechanism has failed; absence of complaints and dispute resolution mechanisms for CSOs and their members; lack of a general framework of principles for collaboration between the government and CSOs [12]. Ghaus-Pasha emphasised the importance of CSOs for promoting local economic development, alleviating poverty, advocating for policy change, contributing to good governance and campaigning for the Millennium Declaration. He asserted that their contributions, however, need to be strengthened and the Millennium Declaration and local civil society movements could strengthen and reinforce each other both at the local and national level [13]. Despite their growing importance, CSOs in the developing world remain only partially understood. Even basic descriptive information about these institutions-their number, size, areas of activity, sources of revenue and the policy framework within which they operate is not available in any systematic way. Moreover, the civil society sector falls in a conceptually complex social terrain that lies mostly outside the market and the state. For much of recent history, social and political discourse has been dominated by the ‘two sector model’ that acknowledges the existence of only two actors in the market: the for-profit private sector and the state. This is reinforced by statistical conventions that have kept the “third sector” of civil society organizations largely invisible in official economic statistics [14].

Despite the existence of many CSOs, some are not active or functional owing to lack of managerial and project implementation capacity and a wider supporting infrastructure for the civil society sector [15]. This is in line with what has been established in this paper. An assessment published by the Raajje Foundation recommended urgent efforts for NGO capacity training, the development of civil society networks and partnerships, and the forging of standards and guiding principles within the civil society sector. Additionally, their findings pointed to the current and potential contribution of civil society towards addressing key governance and national development challenges [16]. This assessment established capacity gaps that require CSOs to be strengthened. Although there is no perfect assessment or evaluation, since there is room for continuous improvement, Rick James retorted that half a loaf of bread is better than none. “Sometimes a quick and dirty evaluation may be better than no evaluation at all” [15]. Based on this information, a number of recommendations can be made for improving CSO performance. Training workshops to address capacity needs and mentoring visits to address organisation specific issues can be implemented, including work plan development, GF related financial management and reporting including the development and use of the GF Financial Manual, and programmatic M & E and reporting using the GF standard tools. There should be periodic capacity assessments to check on actualizing previous recommendations, monitor progress, point out persistent and new gaps and strategize to improve performance of CSOs. Finally a capacity building plan based on the needs of these organisations should be developed to help the CSOs overcome their challenges or shortfalls. Development targets are challenging and demand consistent and sustained financial, technical and human resource inputs, strengthened by intensive and coordinated efforts by all stakeholders. It is only through working together that the public, private sector and NGO communities can increase the effectiveness of their collective drive towards achieving development goals [17].

## Conclusion

In this study, all the CSOs except one were legally compliant and registered with the NGO council. Most CSOs were assessed as average in management capacity, with program management being the best. Despite the weaknesses identified the CSOs had a number of strengths that could be exploited for the success of the grant. Various factors that negatively influenced their performance as well as capacity needs were identified and recommendations to address the issues were made.

### What is known about this topic

- CSOs can be involved in fight against diseases such as malaria;
- Using CSOs can help reach more beneficiaries;
- Stakeholder engagement is key in malaria control.

### What this study adds

- CSOs are different in terms of capacities and experience in CCMM;
- It is critical to assess capacities of CSOs prior to any funding in order to identify the capacity building needs and risks;
- It is a basis of developing capacity buiding plan and mitigation of anticipated risks.
